# Estimating the genetic structure of *Triatoma dimidiata* (Hemiptera: Reduviidae) and the transmission dynamics of *Trypanosoma cruzi* in Boyacá, eastern Colombia

**DOI:** 10.1371/journal.pntd.0010534

**Published:** 2022-07-11

**Authors:** Natalia Velásquez-Ortiz, Carolina Hernández, Omar Cantillo-Barraza, Manuel Medina, Mabel Medina-Alfonso, Sandra Suescún-Carrero, Marina Muñoz, Laura Vega, Sergio Castañeda, Lissa Cruz-Saavedra, Nathalia Ballesteros, Juan David Ramírez

**Affiliations:** 1 Centro de Investigaciones en Microbiología y Biotecnología-UR (CIMBIUR), Facultad de Ciencias Naturales, Universidad del Rosario, Bogotá, Colombia; 2 Centro de Tecnología en Salud (CETESA), Innovaseq SAS, Bogotá, Colombia; 3 Grupo BCEI Universidad de Antioquia, Medellín, Antioquia, Colombia; 4 Programa de Control de Enfermedades Transmitidas por Vectores, Secretaría de Salud Departamental, Tunja, Boyacá, Colombia; 5 Grupo de Investigación del Laboratorio de Salud Pública de Boyacá, Secretaria de Salud de Boyacá, Tunja, Colombia; 6 Molecular Microbiology Laboratory, Department of Pathology, Molecular and Cell-Based Medicine, Icahn School of Medicine at Mount Sinai, New York city, New York, United States of America; Yale School of Public Health, UNITED STATES

## Abstract

Chagas disease is considered a public health issue in Colombia, where many regions are endemic. *Triatoma dimidiata* is an important vector after *Rhodnius prolixus*, and it is gaining importance in Boyacá, eastern Colombia. Following the recent elimination of *R*. *prolixus* in the region, it is pivotal to understand the behavior of *T*. *dimidiata* and the transmission dynamics of *T*. *cruzi*. We used qPCR and Next Generation Sequencing (NGS) to evaluate *T*. *cruzi* infection, parasite load, feeding profiles, and *T*. *cruzi* genotyping for *T*. *dimidiata* specimens collected in nine municipalities in Boyacá and explored *T*. *dimidiata* population genetics. We found that *T*. *dimidiata* populations are composed by a single population with similar genetic characteristics that present infection rates up to 70%, high parasite loads up to 1.46 × 10^9^ parasite-equivalents/mL, a feeding behavior that comprises at least 17 domestic, synanthropic and sylvatic species, and a wide diversity of TcI genotypes even within a single specimen. These results imply that *T*. *dimidiata* behavior is similar to other successful vectors, having a wide variety of blood sources and contributing to the circulation of different genotypes of the parasite, highlighting its importance for *T*. *cruzi* transmission and risk for humans. In the light of the elimination of *R*. *prolixus* in Boyacá and the results we found, we suggest that *T*. *dimidiata* should become a new target for vector control programs. We hope this study provides enough information to enhance surveillance programs and a future effective interruption of *T*. *cruzi* vector transmission in endemic regions.

## Author summary

Chagas disease is a complex zoonotic infection caused by the protozoan *Trypanosoma cruzi*. This pathology is endemic in the Americas and causes a tremendous burden in terms of public health. The feces of triatomine bugs mainly transmit this parasite. A massive diversity of triatomines can be found in the north of South America, where *Rhodnius* is considered the most epidemiologically relevant genus. However, government efforts have attempted to control the vector transmission of specific regions. That is the case of Boyaca in eastern Colombia, which has several municipalities certified as free of *R*. *prolixus* transmission of the parasite. However, other species such as *Triatoma dimidiata* can occupy the left niche due to *R*. *prolixus* elimination. We explored the infection rate, parasite load, feeding preferences, and *T*. *cruzi* diversity in *T*. *dimidiata* specimens collected in municipalities with no *R*. *prolixus* infestation. Our results highlight the preponderant need for increasing serological surveillance and prevention in those communities due to the risk of a plausible reactivation of *T*. *cruzi* vector transmission due to *T*. *dimidiata*.

## Introduction

Chagas disease (CD) is caused by the protozoan parasite *Trypanosoma cruzi* and transmitted by hematophagous insects of the Reduviidae family [1]. This parasite presents a high genetic diversity and has been subdivided into discrete typing units (DTUs): TcI to TcVI and the genotype TcBat (associated with bats). TcI is the most widely distributed in the Americas, the most prevalent in Colombia, and highly diverse based on several molecular markers, where the spliced leader intergenic region of the mini-exon has been used to identify different genotypes (TcIa to TcIe) [2]. Moreover, CD is considered a public health problem in Latin America, especially in Colombia, where it has been estimated that between 700.000 and 1.200.000 people are infected, and 8 million more are at risk of infection [3]. In Colombia, CD is endemic in many geographical regions including Boyacá, Cundinamarca, Casanare, Santander, Norte de Santander and Arauca [4].

*Triatoma dimidiata* is one of the most important vectors of *T*. *cruzi* in Colombia after *Rhodnius prolixus*. It has a wide geographical distribution, can be found in sylvatic and domestic ecotopes, and presents high infection rates with *T*. *cruzi*. Different infection rates with *T*. *cruzi* have been reported in Mexico, Guatemala, Ecuador, Costa Rica, Belize, and Colombia, ranging between 13.7–60% [5–14]. Regarding *T*. *cruzi* genotyping, DTUs reported for *T*. *dimidiata* are TcI (TcIa, TcId), TcII, TcV and TcVI [7,9,15–17]. This species feeds on many hosts, including sylvatic, domestic species, and humans [17]. Due to this behavior, *T*. *dimidiata* contributes actively to the transmission cycle of *T*. *cruzi* across sylvatic and domestic ecotopes [18].

The populations of *T*. *dimidiata* in Colombia differ in many biological, ecological, and epidemiological attributes [19–22], suggesting that there might be micro-evolutionary processes possibly shaped by eco-geographic barriers [18]. This ability of *T*. *dimidiata* to move between sylvatic and domestic ecotopes has also been studied genetically. Genetic markers such as the cytochrome c oxidase subunit 1 (COI) have been used to conduct micro-evolutionary scales studies in *T*. *infestans* locally [23,24,25]. The Internal Transcribed Spacer 2 (ITS-2) and nicotinamide adenine dinucleotide dehydrogenase 4 (ND4) markers were used in *T*. *dimidiata* specimens from six departments of Colombia (Boyacá, Norte de Santander, Santander, Cesar, Magdalena, and Bolivar), showing three gene clusters corresponding to three geographical locations of the country: Caribbean region, Sierra Nevada of Santa Marta and Andean region [26].

Boyacá is located in eastern Colombia and is endemic for Chagas disease; its *T*. *dimidiata* populations are characterized by complex epidemiological cycles as they are found in both sylvatic and domestic ecotopes and present the ability to move between both [18]; thus, increasing the risk of infection for humans. In Boyacá, *T*. *dimidiata* has been found inside houses, evidencing intrusion and colonization processes, contrary to what has been reported in the country’s western region, where this species is not related to domiciliation processes and indeed presents sylvatic habits [27–29]. This is relevant because *T*. *dimidiata* can occupy the niche left by *R*. *prolixus* in Boyacá, where it has been eliminated in 24/123 municipalities between 2014 and 2019 thanks to the “interruption of domiciliary vector transmission of *T*. *cruzi* by *R*. *prolixus*” program [30].

All the aspects described above imply that *T*. *dimidiata* could represent a potential risk for *T*. *cruzi* transmission, especially in places where *R*. *prolixus* is not the primary concern. It is essential to understand the transmission dynamics of *T*. *cruzi* and their vectors behavior to effectively interrupt the parasites’ transmission cycle and to enhance and/or propose new effective and efficient strategies for entomological surveillance and vector control programs. Therefore, we aimed to evaluate the transmission dynamics of *T*. *cruzi* by studying its vector *T*. *dimidiata* at a microgeographic level in nine municipalities of Boyacá, Colombia, in 2019. To accomplish this, we aimed to analyze *T*. *dimidiata* infection rates, parasite load, feeding profiles, and *T*. *cruzi* genotyping (the last two using a Next Generation Sequencing), as well as entomological indices and four genetic markers to explore the variability and genetic differentiation of *T*. *dimidiata* populations.

## Methods

### Ethics statement

Ethical approval (Act No 113 of 2017) was obtained from the Antioquia University’s ethics committee. All infested houses were sprayed with insecticide by SHB for the elimination of triatomine bugs.

### Study area and collection of triatomines

**Fig 1:**
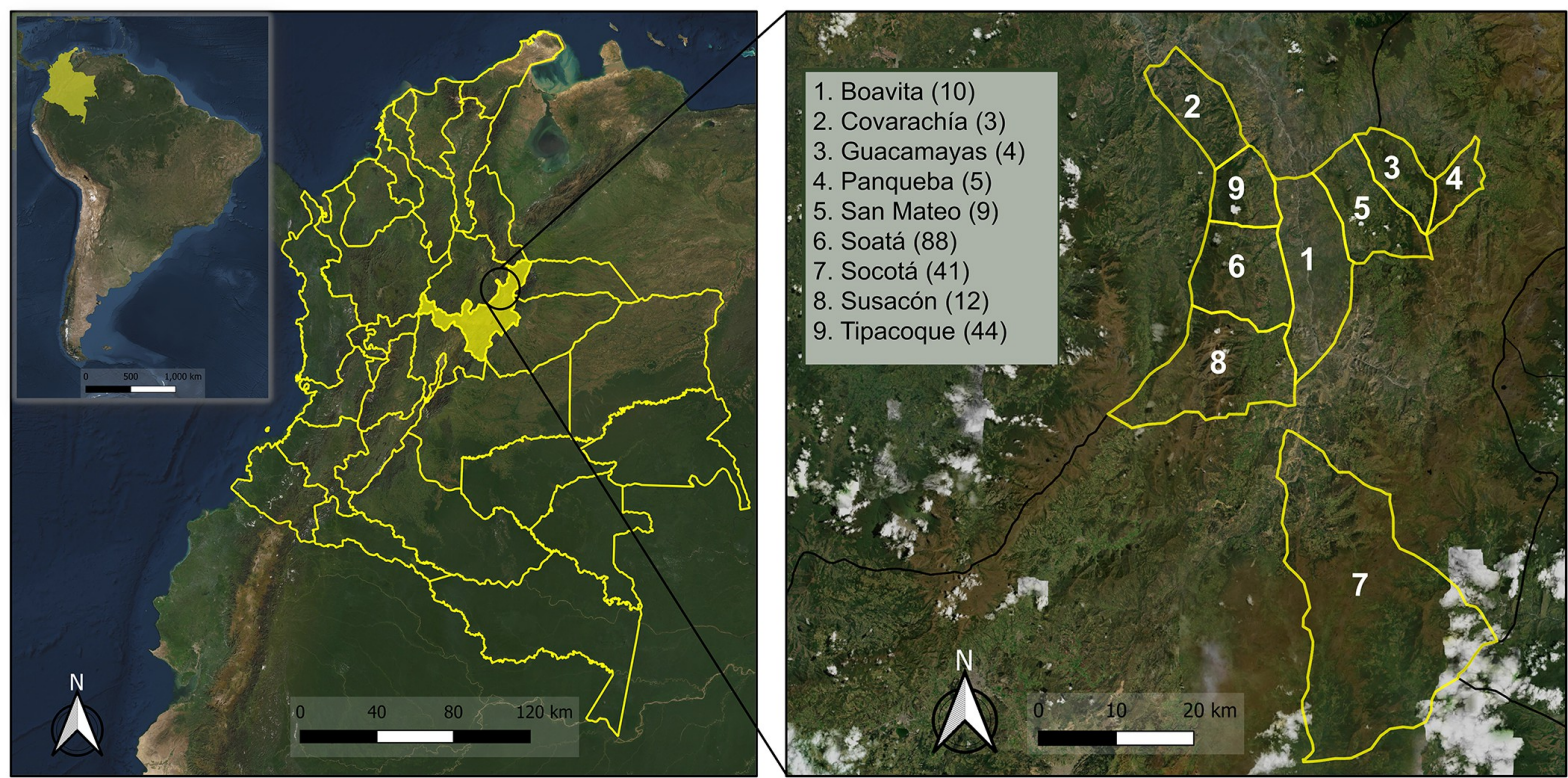
Geographical distribution of collection sites. The map on the left was made with ArcGIS Online [31] (Basemap: Elevation/World_Hillshade https://bit.ly/3vVQ1lL; Sources: Esri, Airbus DS, USGS, NGA, NASA, CGIAR, N Robinson, NCEAS, NLS, OS, NMA, Geodatastyrelsen, Rijkswaterstaat, GSA, Geoland, FEMA, Intermap and the GIS user community). The map on the right was made with QGIS 3.22 (Basemap: Esri Satellite World_Imagery (MapServer) https://bit.ly/3MRtYCf; Sources: Esri, Maxar, Earthstar Geographics, and the GIS User Community; CC BY-SA 3.0) and shows a satellite view of the northern region of Boyacá, Colombia, where the nine municipalities are highlighted in yellow and indicated with numbers. The legend shows the respective municipality and the number of samples collected.

A total of 229 *T*. *dimidiata* specimens were collected in nine municipalities from Boyacá department, Colombia: Boavita (10), Covarachía (3), Guacamayas (4), Panqueba (6), San Mateo (9), Soatá (89), Socotá (45), Susacón (12) and Tipacoque (51) from January to December, 2019 (Fig 1 and S1 Table). The specimens were collected in peridomestic and intradomestic ecotopes. They were identified as adults (male/female) or nymphs, and the collection date was recorded. The 94.32% of the insects (216/229) were collected by residents (passive entomological surveillance), and the remaining 5.68% (13/229) were collected by active entomological surveillance. All the collected insects were sent to the PITs (Triatomine Information Stations- acronym in Spanish) located in each municipality. Samples were stored in plastic and glass containers labeled with the collection date, municipality, and ecotope (peri or intradomicile) and sent to the Department Health Laboratory of Boyacá for taxonomic identification.

### Molecular characterization and population genetics of *T. dimidiata*

In order to characterize the genetic populations of *T*. *dimidiata*. DNA of the insects was obtained from the abdomen. DNA extraction was conducted using a DNeasy Blood and Tissue Kit (Qiagen, Hilden, Germany). Two mitochondrial and two nuclear molecular markers were amplified. For the mitochondrial markers, a 624 bp fragment of the ND4 mitochondrial molecular marker was amplified by PCR as previously described [21,26,32], using the primers ND4-F (5’- TCAACATGAGCCCTTGGAAG- 3’) and ND4-R (5’- TAATTCGTTGTCATGGTAATG- 3’) [21,26]; and a 522 bp fragment of the Cytb mitochondrial gene using primers CYTB7432F (5′- GGACGWGGWATTTATTATGGATC -3’) and CYTB7433R (5′- GCWCCAATTCARGTTARTAA 3’) [33]. For the nuclear markers, the ITS-2 (252 bp) and 28S (696 bp) rDNA genes were amplified using the primers TdITSF (5’-TGGAAATTTTCTGTTGTCCACA- 3’) and TdITS-2R (5’-CTTGCTTTATACAACAAGAAGTA- 3’) and D2F (5′- GCGAGTCGTGTTGCTTGATAGTGCAG- 3′) and D2R (5′-TTGGTCCGTGTTTCAAGACGGG- 3′), respectively as described previously [26, 34]. PCR products were cleaned using ExoSAP-IT and sequenced by Sanger. A total of 144 amplicons were sent for sequencing (36 samples per marker) to avoid overrepresentation because some municipalities had few specimens (Covarachía (3) and Guacamayas (4)) than others, such as Soatá (89) or Socotá (45). For the municipalities with more than four specimens, we randomly selected a maximum of 5 specimens, ensuring we did not choose them from the same dwelling.

The sequences obtained for each molecular marker were aligned using Seqman (DNASTAR Lasergene 7) and manually checked for misalignments (GenBank accession codes ON323595-ON323660). Colombian GenBank sequences for each marker were included in the phylogenetic analyses (S2 Table). The alignments of the GenBank and herein obtained sequences were made using the MUSCLE algorithm in UGENE software [35]. Haplotype networks were constructed using POPART 1.7 Median Joining method (MJ) [36], and maximum likelihood phylogenetic analyses were performed using IQ-TREE v1.6.12 [37]. The trees were constructed for each genetic marker and visualized in iTOL v6 [38]. Ultrafast bootstrap approximation with 10,000 repetitions was carried out for branches support. Lastly, for each marker were calculated summary population genetics statistics and haplotype data (number of haplotypes (h), polymorphic sites (S), nucleotide diversity (π), haplotype diversity (Hd), Tajima’s D, Fu & Li’s D* and F*) (Table 1) to estimate the genetic diversity of *T*. *dimidiata* Boyacá populations and F_st_ to estimate their gene flow using the software DnaSP v6 [39].

**Table 1:** Haplotype and polymorphism data analysis summary for Boyacá sequences.

	Cytb	ND4	28S	ITS-2
Sequence length	510	565	573	304
number of sequences	30 (34)^a^	26 (36)	25 (36)	32 (36)
Polymorphic sites (S)	43	222	5	0
h	15	17	11	1
Hd	0.9011	0.9231	0.9000	0.0000
Pi	0.01952	0.10323	0.00332	0.0000
Tajima’s D	-0.32628 p > 0.10 no sig	-1.52558 p > 0.10 no sig	0.45991 p > 0.10 no sig	0.0000
Fu and Li’s D*	0.03664 p > 0.10 no sig	1.33617 p > 0.10 no sig	0.48652 p > 0.10 no sig	0.0000
Fu and Li’s F*	-0.09706 p > 0.10 no sig	0.47998 p > 0.10 no sig	0.55576 p > 0.10 no sig	0.0000

^a^Two sequences for cytb were unable to assemble due to sequencing quality and therefore discarded from the analysis.

### Molecular detection and genotyping of *T. cruzi*

A quantitative PCR was conducted for the detection of *T*. *cruzi*. For detection and amplification, it was used a TaqMan Fast Advanced Master Mix 2× (Roche Diagnostics GmbH, Mannheim, Germany), water, and the primers cruzi1 (0.75 μM) (5′-AST CGG CTG ATC GTT TTC-3′), cruzi2 (0.75 μM) (5′-AAT TCC TCC AAG CAG CGG ATA-3′) and a cruzi3 probe (0.05 μM) (FAM-CAC ACA CTG GAC ACC AA-NFQ-MGB) to detect the satellite tandem repeat DNA of the parasite (166 bp) following the conditions previously reported [40]. As reported elsewhere, a Ct value < 38 was considered positive [5].

A standard parasite curve was prepared for quantification using *T*. *cruzi* MG (TcI) strain metacyclic trypomastigotes obtained as reported previously [41], that were isolated and quantified using a Neubauer chamber to obtain 10^11^ parasites per mL. The abdomen section of non-infected adult *R*. *prolixus* specimens were used to spike them with metacyclic trypomastigotes; then, 500 μL of the previously resuspended solution of the dissected abdomen was spiked with 10^11^ parasites to perform the disruption step, where both parasites and insects abdomen were shredded in FastPrep lysis beads and matrix tubes for sample disruption with lysis buffer. Subsequently, proteinase K was added and samples were placed in incubation at 56°C and 200 rpm for 12–14 hours. Then, using DNeasy mini spin columns on Eppendorf tubes, DNA was recovered from the shredded mix obtained from the disruption process using the elution buffer. Samples were quantified using Nanodrop and stored at -20°C. All the DNA extraction procedures were conducted using the DNeasy Blood and Tissue Kit (Qiagen, Hilden, Germany).

After DNA extraction, serial dilutions from 10^11^ to 10^−1^ of the DNA were made with DNA obtained from the abdomen of non-infected *R*. *prolixus* specimens and stored at -20°C. A qPCR was run with the standard curve (serial dilutions) placed by duplicate in every qPCR plate with the samples to get the number of parasites. Lastly, sample amplification was checked, verifying the no amplification of negative controls and correct amplification of the parasites curve. The quantity mean of parasites per sample was calculated automatically using the linear regression equation of the standard curve of parasites made by the Real-Time Software Quant Studio Design and Analysis (Applied Biosystems).

Parasite genotyping was accomplished by conventional PCR, amplifying the spliced leader intergenic region of the miniexon gene (SL-IR) that divides DTUs into two groups: TcI (350 bp) and TcII-TcVI (300 bp). The reaction mix contained Go Taq Green Master Mix 1×, water, and primers TCC (1.25 nM) (5′-CCC CCC TCC CAG GCC ACA CTG-3′), TC1 (1.25 nM) (5′-GTG TCC GCC ACC TCC TTC GGG CC-3′) and TC2 (1.25 nM) (5′-CCT GCA GGC ACA CGT GTG TGT G-3′) [42]. A random selection of positive samples (27) was submitted to Illumina NovaSeq 6000 platform for sequencing (Novogene), paired-end 150 bp with sequencing depth above 200x. Samples that failed quality requirements for Illumina (6/27) were sent to Sanger sequencing. Illumina sequencing products were passed through quality control (FastQC [43], MultiQC [44]) to discard sequences with incongruences or low quality. Then, QIIME [45] was used to remove the barcodes when necessary. SL-IR miniexon gene data is deposited in the European Nucleotide Archive (ENA) at EMBL-EBI under the accession number PRJEB52453. Clean sequences were submitted to BLASTn for similarity search (96% of identity, e-value 10) and compared against a reference dataset constructed using GenBank sequences of TcI genotypes sequences previously reported in Colombia [46] and reference sequences from TcII to TcVI and Tcbat (S3 Table). DADA2 pipeline was used to keep track of how many times each specific amplicon sequence variant (ASV) was found in each sample. The number of reads in the ASV output table was converted to relative values and grouped by sample using RStudio so it can be used as an approximation of each TcI genotype abundance per sample. Then, a barplot of relative abundance was made with ggplot2 package [47].

### Molecular characterization of feeding sources

The DNA of randomly selected samples positive for *T*. *cruzi* (27) were submitted to Illumina NovaSeq 6000 (Novogene Co., Ltd) for sequencing of a 12S rRNA gene fragment (215 bp). Samples that failed quality requirements for Illumina (9/27) were sent to Sanger sequencing. Illumina sequencing products were passed through quality control (Fast QC, MultiQC) to discard sequences with incongruences or low quality. Then, QIIME was used to remove the barcodes when necessary. The data of 12S rRNA gene is deposited in the European Nucleotide Archive (ENA) at EMBL-EBI under the accession number PRJEB52450. Sequences were submitted to BLASTn for similarity search and compared against a reference dataset with curated sequences as described before [48]. A manual check of the animal distribution in Colombia was made using the following web pages: www.iucnredlist.org, www.eBird.org, and www.colombia.inaturalist.org. The three datasets were merged into one extensive dataset with 137 sequences of vertebrates. The final dataset was uploaded to Github (https://github.com/nvelasquez30/Triatoma_dimidiata_food-sources_12SrRNA). The number of reads per vertebrate was used to approximate its abundance in the triatomine diet [17]. DADA 2 pipeline and relative abundance barplot were also performed and made as described in the previous section.

### Statistical analysis

Kolmogorov-Smirnov normality test evaluated any associations between parasite load and other variables (ecotope, insect collection month, development stage, and municipality). Non-parametric Wilcoxon tests were made to compare between sample groups. Hence, a post hoc analysis (Dunn test adjusted with the Bonferroni method) was conducted to correct the analysis per sample size. Additionally, a Shapiro-Wilk normality test was performed to evaluate if there was any relation between the triatomine blood meals and the parasite load. A Wilcoxon rank-sum exact test was then executed to compare the relative abundance of the most common blood meals with the parasite load, and finally, to assess correlation, a Spearman test was made. For this test, it was used the dataset with the relative abundances. All statistic tests were run in RStudio v4.1.2 with the package Rcmdr v2.7–2.

The entomological indices estimated were the following five according to the WHO guidelines for Chagas disease Control [49]: Infestation (number of houses infested by triatomines/number of houses examined)x100, density (number of triatomines captured/number of houses examined), crowding (number of triatomines captured/number of houses with triatomines), colonization (number of houses with triatomine nymphs/number of houses positive for triatomines)x100 and infection (number of infected triatomines/total of triatomines captured)x100. These indices are estimated to compile information about the vector in specific zones and evaluate the possible impact of future control interventions [50].

## Results

### *Triatoma dimidiata* population genetics

Once the Sanger sequences were obtained, a polymorphism data analysis was made using DnaSP for each genetic marker (cytb, ND4, 28S, ITS2). For ND4 mitochondrial marker, it was found the majority of haplotypes (h) with 17, followed by cytb with 15, 28S with 11, and ITS2 with 1. Regarding the haplotypic diversity (Hd) values, ND4, cytb, and 28S presented values of 0.9, slightly higher for ND4, and nucleotide diversity (π) of 0.1, 0.01, and 0.003, respectively. Neutrality and demographic history tests, such as Tajima’s D and Fu & Li’s D* and F*, showed no significative p values >0.10 (Table 1).

No further analyses were made because the haplotype, polymorphism, and phylogenetic data showed no significant changes for cytb, 28S, and ITS-2 (Table 1 and S1 and S2 Figs). On the other hand, for the mitochondrial markers and subsequent analyses, ND4 was chosen as it has shown a better resolution to study specifically *T*. *dimidiata* populations [26].

**Fig 2:**
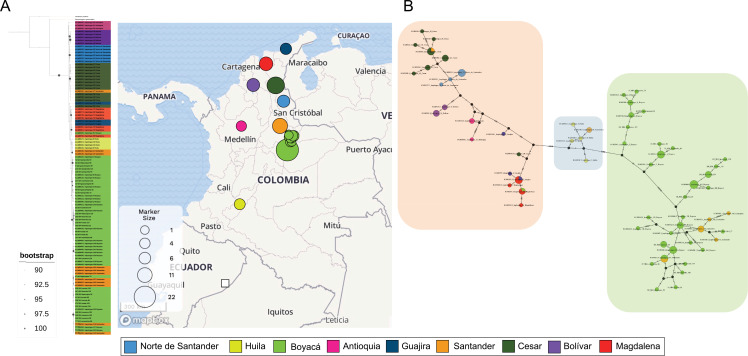
Maximum Likelihood phylogenetic reconstruction and haplotype analyses for ND4 genetic marker. A) shows the phylogenetic relationship between ND4 sequences obtained from GenBank (74) and in the present study (33). The map was made using Microreact [51] Maps Mapbox (www.mapbox.com/about/maps) and OpenStreetMap (www.openstreetmap.org/about). Circles’ size in the map is related to the number of sequences used for the analysis. The tree was rooted with *Rhodnius prolixus* and *Panstrongylus geniculatus* mitochondrial partial sequences. B) Haplotype network of ND4 sequences. Green box groups (Boyacá and Santander haplotypes), blue box groups (Huila haplotypes (southwest)), and orange box groups (the haplotypes belonging to northern Colombia).

For ND4, phylogenetic analyses showed that the new sequences were clustered together with GenBank Boyacá sequences, and these at once define a single cluster, including those from another geographical region in Colombia (Figs 2A and S3). Haplotype network analysis showed that it seems the haplotypes are to some extent grouped by geographical region: Boyacá and Santander haplotypes, Huila haplotypes (southwest), and one last group containing sequences from the other regions that belong to the northernmost part of Colombia (Antioquia, Norte de Santander, Bolívar, Cesar, Magdalena, and Guajira) (Fig 2B). A similar case occurred with cytb and 28S, where phylogenetic trees showed a single cluster and the haplotype networks patterns were homogeneous, revealing no clear genetic structure (S1 and S2 Figs, respectively). Finally, ITS2 phylogeny and haplotype network were not informative, considering it has a single haplotype.

Besides, a genetic differentiation analysis was performed using DNASP solely for the ND4 sequences obtained in this study. Sequences sets were defined per municipality, resulting in 8 populations (Covarachía was excluded for sampling). F_st_ results of paired comparisons between population/municipality sequences were obtained: Guacamayas vs Socotá, Soatá, Boavita and Susacón = 0.719, 0.715, 0.708 and 0.757, respectively; Socotá vs Panqueba, Soatá and Boavita = 0.747, 0.483 and 0.472; Panqueba vs Soatá, Boavita and Susacón = 0.727, 0.726 and 0.779; Soatá vs Susacón = 0.567; and Boavita vs Susacón = 0.617.

### *T. cruzi* infection, parasite load and DTUs

**Fig 3:**
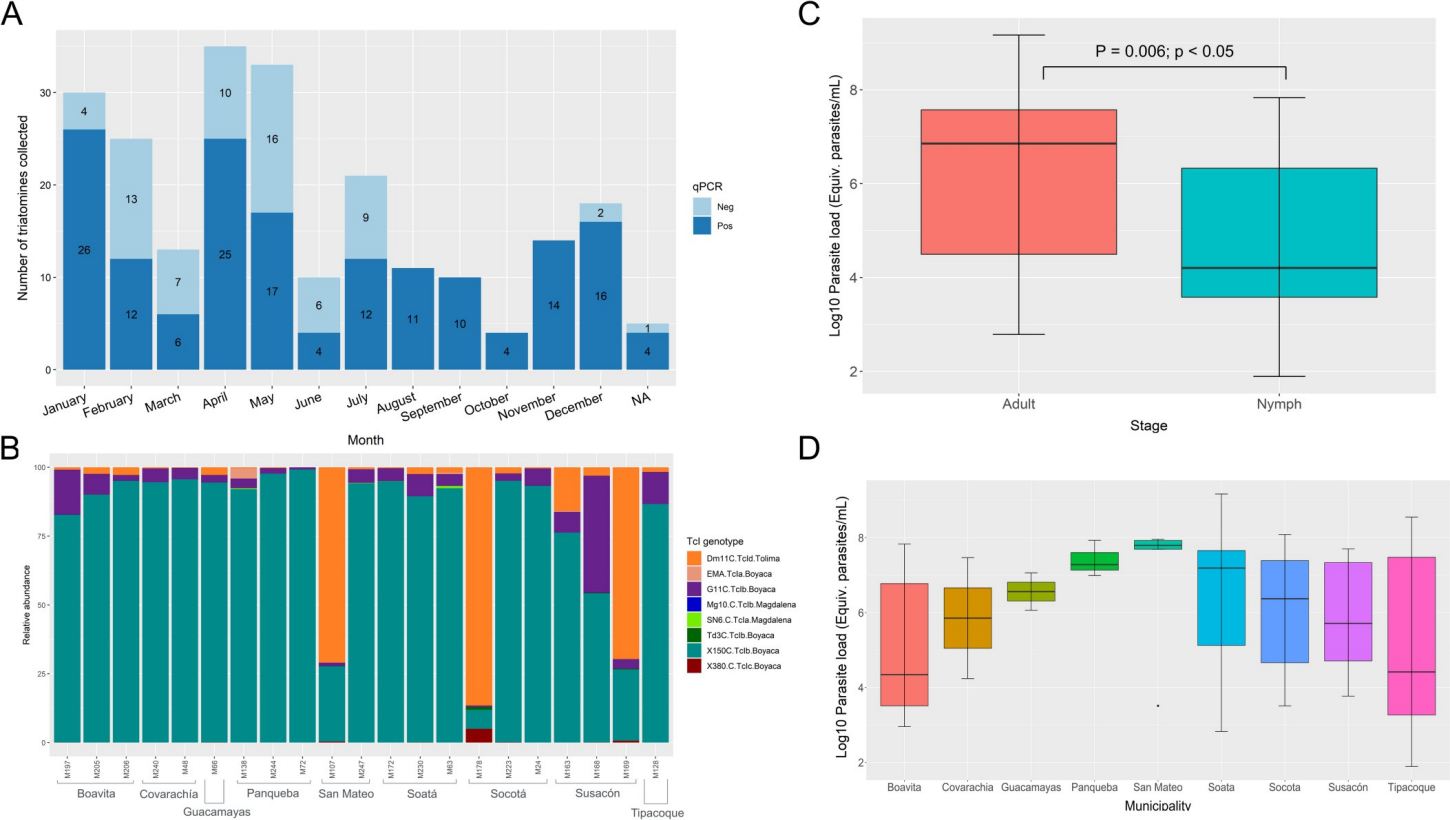
*T*. *cruzi* infection, DTUs, and parasite loads of *T*. *dimidiata* specimens collected. A) Number of triatomines collected per month and infection qPCR results (positive or negative), B) Relative abundance of TcI genotypes per sample and municipality where each sample was collected, C) Parasite loads values per developmental stage (p-value of 0.006; p<0.05) and D) Parasite loads per municipality (p-value: 0.048; p<0.05). Boxplots parasite load values on Y axis were transformed to Log10 scale to enhance the data visualization considering the outlier loads we observed.

A total of 161 *T*. *dimidiata* specimens collected (70.31%; 161/229) were positive for *T*. *cruzi* infection. Of these, DTU TcI was detected in 83.85% (135/161) of the insects, the only DTU found. The remaining percentage of samples could not be genotyped by conventional PCR. Fig 3A shows a constant presence throughout the year of triatomines in the dwellings, most of them infected. As a new approach, TcI genotyping was achieved using NGS (Illumina NovaSeq 6000), obtaining the relative abundance (estimated by the reads number) of the different TcI genotypes present in one sample (Fig 3B). Here, TcIb was the most common genotype in the samples, with three exceptions where TcId was predominant. Also, the TcIc genotype was detected in some samples. Likewise, we detected some samples that contained two or more genotypes simultaneously.

Regarding the parasite load, the comparison between the parasite load by development stage was statistically significative for the adult stage with a p-value of 0.006 (p<0.05), indicating parasite load is higher in adults than nymphs (Fig 3C), and the comparison among parasite load vs. municipality was 0.048 (p<0.05). The boxplot, in this case, showed that the municipalities of San Mateo and Panqueba presented a higher parasite load (Fig 3D). The Dunn test showed no statistically significant differences of parasitic load per municipality (p-values > 0.1), although some comparisons had a p-value different from 1, such as Boavita-San Mateo (p = 0.55), San Mateo-Tipacoque (p = 0.33) and Soatá- Tipacoque (p = 0.15).

### *T. dimidiata* blood meals

NGS sequencing of the 12S marker confirmed the whole gamma of hosts *T*. *dimidiata* could feed on (S4 Fig), where human (*Homo sapiens sapiens*) is the most common, followed by the dog (*Canis lupus familiaris*) and cow (*Bos taurus*). Even some birds such as hen (*Gallus gallus*), duck (*Cairina moschata*) and Black vulture (*Coragyps atratus*), marsupials such as opossums (*Didelphis marsupialis* and *Metachirus nudicaudatus*), rodents such as house mouse (*Mus musculus*) and rat (*Rattus rattus*), bats such as *Carollia perspiscillata* and *Sturnira tildae*, and other mammals such as buffalo (*Bubalus bubalis*), donkey (*Equus asinus*), cat (*Felis catus*), sloth (*Choloepus didactylus*) and pig (*Sus scrofa domestica*) (S4 Fig).

**Fig 4:**
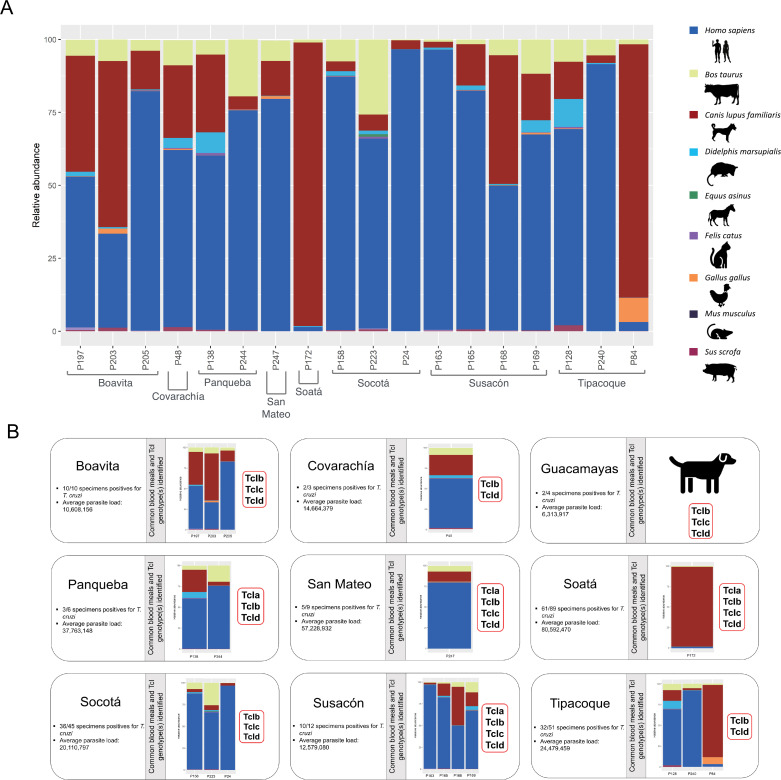
Summary of infection, parasite loads, feeding profiles, and TcI genotypes information of *T*. *dimidiata* specimens per municipality. A) The barplot shows the top 9 most common blood meals *T*. *dimidiata* fed on, the hosts cartoons were made by the authors. B) Each inset contains information per municipality, such as average parasite load (total samples collected per municipality), infection data, common blood meals, and TcI genotype identified per sample. Guacamayas’ sample was sequenced by Sanger; therefore, it does not appear in the barplot, but blood meal information is represented in the inset. Complete information of sequenced samples available in S4 Table.

A bar plot of the top 9 most common blood meals was made to facilitate the analysis. Fig 4 multipanel shows the barplot and merged information per municipality that comprises a blood meal barplot including the sequenced samples, their respective TcI genotypes, infection, and average parasite load (of the samples per municipality). Guacamayas’s samples were sequenced by sanger (samples failed quality controls for Illumina Novaseq). In general, it can be observed that *T*. *dimidiata* individuals in each municipality feed on various animals and transmit domestic-peridomestic (TcIa, TcIb, TcIc) and sylvatic associated (TcId) genotypes. To evaluate if there is a relation between the parasite load and blood meals, a Shapiro-Wilk normality test was performed for parasite load, and the data does not have a normal distribution (p<0.05). Wilcoxon test results showed no statistically significant differences (p > 0.05). A Spearman correlation test was made to compare the parasite load versus the relative abundance values for each animal, but no statistically significant correlations were found (p > 0.05).

### Entomological indices

The entomological indices per municipality were calculated considering the collection type, but only the insects captured by the residents were used for analysis (passive surveillance—PS). S5 Table shows the infestation, colonization, density, crowding, and infection indices. Broadly, it can be observed that Boavita, Panqueba, Socotá, and Susacón presented the highest infection indices (100%, 75%, 87.5%, 81.8%, respectively). Susacón had the highest crowding index value (6), which means this municipality presented the most considerable amount of captured triatomines in fewer houses. The density index shows Boavita (3) and Socotá (3.3) were the municipalities with the most triatomines collected per house examined. The infestation index indicates that Boavita, Panqueba, San Mateo, and Socotá presented the highest percentage of triatomines captured in the houses (100%, 75%, 100%, 83.3%, respectively). Lastly, the colonization index shows that the houses in the nine municipalities are infested with nymphs in the range between 20% and 66.7%.

## Discussion

The effectiveness of vector control programs greatly depends on studying their population structure; thus, macro and micro geographical genetic analyses are crucial to understanding migration patterns, evolutionary history, and events of recolonization and reinfestation by triatomines [50]. Considering the recent vector transmission elimination of *R*. *prolixus* was achieved in some municipalities, studies focused on the description of *T*. *dimidiata* -and other triatomines considered secondary vectors- behavior must be prioritized to minimize the risk of Chagas disease transmission in Boyacá and surroundings. To further address this, we first evaluated the genetic diversity of *T*. *dimidiata* in several municipalities of Boyacá. We found that the ND4 marker had the most polymorphic sites, presenting a higher number of polymorphisms [21]; therefore, higher Hd values and the number of haplotypes were obtained (Table 1). Similar results for ND4 in *T*. *dimidiata* were found among 3 Colombian departments (Guajira, Cesar, and Santander) using ND4 as the molecular marker (Hd = 0.863 ± 0.048 and π = 0.034 ± 0.003) [21]. Then, we wanted to evaluate the genetic differentiation, we did not find significant F_st_ values, and the topologies of the phylogenetic trees and patterns of the haplotype networks (Figs 2, S1, and S2) showed a single cluster containing all the samples. Based on the results, we suggest that *T*. *dimidiata* populations in Boyacá at least for the ND4 marker conform a similar population with no explicit evidence of differentiation. The application of treatments for vector control in Boyacá for *T*. *dimidiata* could have the same or similar results as the populations have similar genetic characteristics, but further studies must be done before and after insecticide spraying to evaluate this possibility. Even though, the success of fumigations will depend on other factors such as the period of spraying, the insecticide type, and their residual effects [52]. Knowing that *T*. *dimidiata* in Boyacá comprise a single population, vector control programs can be planned as its populations are similar, and a single protocol might be effective in the department. Nevertheless, it is essential to perform more studies comprising more municipalities in the North region of Boyacá to confirm that the same pattern is also kept along the department.

Regarding *T*. *cruzi* infection, in a recent study in Boyacá between 2017 and 2018, it was found that *T*. *dimidiata* presented the highest natural infection index (37.8%) [30]. In other regions, such as Santander, *T*. *dimidiata* present an infection rate of 33% [52]. We found an infection rate of 70.31% in 2019, slightly more than double what was reported for the preceding years in the same department [30]. It is important to highlight that the result we obtained for *T*. *dimidiata* are similar to values reported for other epidemiologically relevant triatomine species in the Americas (also including some secondary vectors): *Rhodnius prolixus* (73.3%, 55.8%) [5,48], *Panstrongylus geniculatus* (56%, 87.5%, 70.6%) [5,48,53], *Triatoma maculata* (67.6%) [5], *Rhodnius pallescens* (45.9%) [5], *Rhodnius pictipes* (87.5%) [4], *Mepraia spinolai* (14–76%) [54,55] and *Triatoma brasiliensis* (peridomestic: 36–71%, sylvatic: 28–100%) [56]. This implies that *T*. *dimidiata* might represent a risk for *T*. *cruzi* transmission because they move around significantly, feeding on many domestic and sylvatic reservoirs (as we will discuss later) where they obtain the parasite, which might represent a higher risk for humans.

One of the most important results we obtained was the report of the first parasitic load data for *T*. *dimidiata* naturally infected, as it is an important epidemiology variable to consider when evaluating the risk of transmission of *T*. *cruzi*. We standardized a quantitative PCR (qPCR) and made a parasite standard curve using metacyclic trypomastigotes at a concentration of 10^11^. We found loads as large as 1.46 × 10^9^ parasite-equivalents/mL. Similar values have been found for *Mepraia spinolai*, the main vector in Chile (1.29 × 10^9^ par-eq) [57]. In addition, we found the parasite load was statistically significative higher in adult specimens than in nymphs (p = 0.006; p<0.05) and per municipality (p = 0.048; p<0.05) (Fig 3C and 3D). Our results indicate that *T*. *dimidiata* harbors a significant quantity of parasites that could represent an important risk for *T*. *cruzi* transmission because the parasites’ quantity can be determinant for the morbidity and development of the Chagas disease [58]. Even though, the amount of metacyclic trypomastigotes present must also be considered as they are the infective forms of the parasite. Moreover, parasite transmission depends on other factors related to the vector capacity of triatomines [59] that should be evaluated for *T*. *dimidiata*. Future studies are needed to evaluate the vector capacity of *T*. *dimidiata* thoroughly, considering animal models for research, and cutting-edge methodologies like the one herein described [60]. Further studies must be done to evaluate the parasitic load on the hosts and evaluate if its relationship with the parasite load depends on the host or the number of times *T*. *dimidiata* fed on. Indeed, we tried to address that issue by comparing the parasite load vs. the relative abundances for each blood meal, but we found no significant differences. We know that the sample size might have influenced the results of the tests, although similar conclusions from this comparison were obtained for *Mepraia spinolai* [57]. This, in the end, reinforces the pivotal need to unveil the ecology of *T*. *dimidiata* at local and regional scales.

About the feeding behavior, we found that *T*. *dimidiata* fed on at least 17 animal species among domestic, synanthropic, and sylvatic species (S4 Fig), as reported before [5,17], confirming the vast diversity of hosts this triatomine has. We reported some new blood meals, for one of them, the buffalo (*Bubalus bubalis*), there are no reports of species of *Trypanosoma* other than *Trypanosoma vivax* [61], which causes cattle trypanosomiasis [62]. This new discovery is important as *B*. *bubalis* can be a new intermediary host for *T*. *cruzi*, but more studies are needed. These results suggest that *T*. *dimidiata* could play an essential role in the transmission of domestic and sylvatic *T*. *cruzi* genotypes [17], but future studies must include active search of intradomicile nymphs and sylvatic individuals for analysis. Also, it shows a behavior similar to other essential vector species, like *R*. *prolixus* and *P*. *geniculatus*, having the same variety of feeding sources, with humans as the most common among them, followed by dogs, cows, and mice [5,17,48]. A study recently made showed *T*. *dimidiata* presented the most quantity of feeding sources with 18, followed by *T*. *maculata* with 11 and *R*. *prolixus* with 9, sharing *Homo sapiens*, *Canis*, *Felis*, *Bos*, *Sus*, *Didelphis*, and *Metachirus* [63], herein identified as well (S4 Fig). These results imply that *T*. *dimidiata*’s feeding behavior is highly similar to other successful vectors, evidencing a parasite’s transmission cycle encompassing the broadest range of hosts and vectors that do not support a strict domestic/sylvatic transmission cycle [63]. This highlights the importance of *T*. *dimidiata* as a vector, especially in areas where other primary species have been controlled, also considering this species moves constantly across long distances [30]. This should be an essential concern in future discussions of vector control across Boyacá and eastern Colombia.

Regarding the DTUs, TcI was the only found, as reported before, in *T*. *dimidiata* [63,64]. With the NGS strategy, TcIb was the most common, its presence has been confirmed in Colombia, Costa Rica, and Mexico before (Fig 3B) [62]. We also found a combination of TcIa, TcIb, TcId, and even TcIc (Fig 3B) associated with domestic-peridomestic transmission cycles overlapping [65]. The genotype TcIc has been reported before in Boyacá in *T*. *dimidiata* and *R*. *prolixus*, confirming there is no change in the circulating genotypes following the elimination of *R*. *prolixus* [65]. Also, it can be noted that even though most of the blood meals were domestic animals, sylvatic genotypes (TcId) are detected, suggesting again an overlap of *T*. *cruzi* transmission cycles thanks to the movement of *T*. *dimidiata* and the interaction with the hosts/reservoirs. The above suggests that *T*. *dimidiata* is constantly moving/walking across ecotopes [66], probably contributing to the parasites’ transmission cycle and intrusion of sylvatic genotypes into the domestic cycle. The sylvatic genotype (TcId) has been related to cardiac histiotropism. In contrast, domestic genotypes (TcIa) are primarily found in the bloodstream [42,67]. Then, our findings highlight the need for this type of studies to assess the risk to the residents that are exposed in case they get infected, considering multiple *T*. *cruzi* genotypes could influence host immune response and modulate the progression of the disease [68].

We finally calculated the five entomological indices (infestation, colonization, density, crowding, and infection) for the nine municipalities using the passive surveillance data (S5 Table). Boyacá and Santander have traditionally been departments with the highest indices of infestation, infection, colonization, and dispersion by *T*. *dimidiata*. Here, at a local level, we found infestation indices ranging from 50% to 100% and crowding indices from 2 to 6. These two indices together can indicate more risk of a person getting infected with the parasite as many infected triatomines can feed on the same host, increasing the risk of transmission. In this scenario, for the people from municipalities such as Susacón, Socotá, and Boavita, there should be Chagas disease screening controls and active vector searching in their houses to reduce the infestation and crowding indices. Many factors should be considered when applying these indices for vector control strategies, like the month of the year and climate variables, as domicile occurrence of the triatomines can be influenced by specific climatic conditions [69]. In this case, we found that the triatomines are reaching the dwellings throughout the year, and considering their infection rates and feeding behavior, there might be constant rather than occasional transmission of *T*. *cruzi* (Fig 3A). Some studies in South and Central America reported that the arrival and abundance of triatomines are higher in the dry season than in the rainy season [70–75]. In Boyacá, the dry season happens twice a year at the beginning and middle of the year, while the rainy season happens in April-May and October-November [76]. Our results did not agree at all with this seasonality pattern -that in other vectors like *R*. *prolixus* indeed happen in Casanare, Colombia [77]-, but we can associate the occurrence of *T*. *dimidiata* in rainy months with the fact that inside or close to the houses they can find both blood sources and shelter to survive. This can be important for colonization indices as *T*. *dimidiata* can frequently arrive at the houses and start a domiciliation process, as noted for the evaluated municipalities with colonization indices among 20% and 100% (S5 Table). Nevertheless, an active search of nymphal stages in the houses must be made to remove or prevent the colonization.

Our results confirmed the vast diversity of blood sources *T*. *dimidiata* can have, their constant movement across ecotopes and their contribution to the circulation of sylvatic genotypes into the domestic cycle of TcI. This analysis by the municipality allows the implementation of specific strategies for vector control as local knowledge is essential and can be used to influence vector control. Also, even though our results did not agree with the occurrence (frequency of arrival) and density (amount) of triatomines inside and near the houses by seasonality, it is essential to consider the monthly occurrence to know the moment when intervention control is allowed [77]. Moreover, as there is a single population of *T*. *dimidiata* circulating in Boyacá, vector control programs can be planned more effectively to avoid resistance or reinfestations, as it already happened in Nicaragua and Guatemala, where the vector control program failed to suppress house reinfestation by *T*. *dimidiata* [78,79]. Last but not least, with the parasite loads results we obtained, considering the most common blood source was humans and the presence and density of triatomines inside the houses all year long, we emphasize the need for serological studies of the population inhabiting these municipalities. All these results highlight that despite *R*. *prolixus* being successfully eliminated, *T*. *dimidiata* must become the new target for the government stakeholders and vector control programs.

Hopefully, this study can provide insights on the enhancement of vector control programs, and we encourage this type of studies to be carried out frequently so an effective interruption of *T*. *cruzi* vector transmission can be achieved, especially in endemic departments for Chagas disease that can be extrapolated to other regions in the Americas. The combination of ecology, molecular biology, and next-generation sequencing has been pivotal for a better comprehension of *T*. *cruzi* transmission dynamics. Further research must include better sampling in more locations as possible and serological studies of the residents to have a close-up of the risk to which they are exposed. After all, the improvements mentioned above can help to avoid failed vector control programs and make them sustainable in the longer term.

## Supporting information

S1 Fig Phylogenetic tree and haplotype network for cytb genetic marker. Purple bars on the left indicate the number of haplotypes each sequence had.
(TIF)

S2 Fig Phylogenetic tree and haplotype network for 28S genetic marker. Green bars on the left indicate the number of haplotypes each sequence had.
(TIF)

S3 Fig Phylogenetic tree and haplotype analyses for ND4 marker without GenBank sequences. The tree shows the evolutionary relationships among the sequences obtained in this study. Legend follows the same color code per municipality used for the phylogenetic reconstruction.
(TIF)

S4 Fig Relative abundance of *T*. *dimidiata* blood meals per sample. (TIF)

S1 Table Summary table that contains information from the 229 triatomines analyzed. (DOCX)

S2 Table GenBank accession codes for the molecular markers used in the phylogenetic analyses. (DOCX)

S3 Table GenBank accession codes of the sequences used for the construction of the reference DTUs dataset for BLAST similarity search. (DOCX)

S4 Table Summary table that contains sequenced samples crossed information. (DOCX)

S5 Table Summary of entomological indices per municipality. (DOCX)
