# Supplementary figures and images for: Estimating the genetic structure of Triatoma dimidiata (Hemiptera: Reduviidae) and the transmission dynamics of Trypanosoma cruzi in Boyacá, eastern Colombia
Source: PLoS Negl Trop Dis. 2022 Jul 11;16(7):e0010534. doi: 10.1371/journal.pntd.0010534 (PMC9302734; doi:10.1371/journal.pntd.0010534)

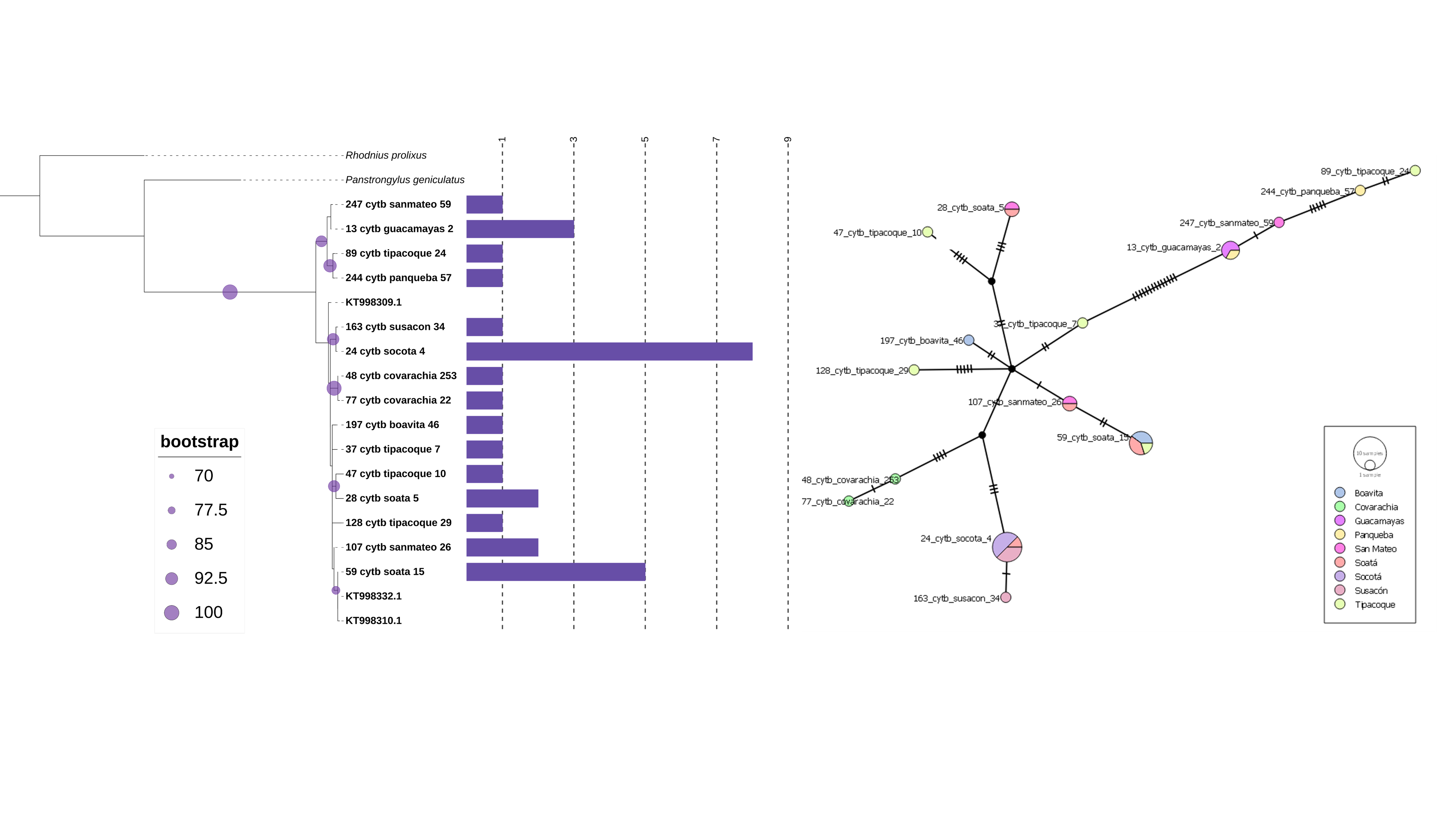

Supplement: S1 Fig — Purple bars on the left indicate the number of haplotypes each sequence had. (TIF) [file pntd.0010534.s001.tif]

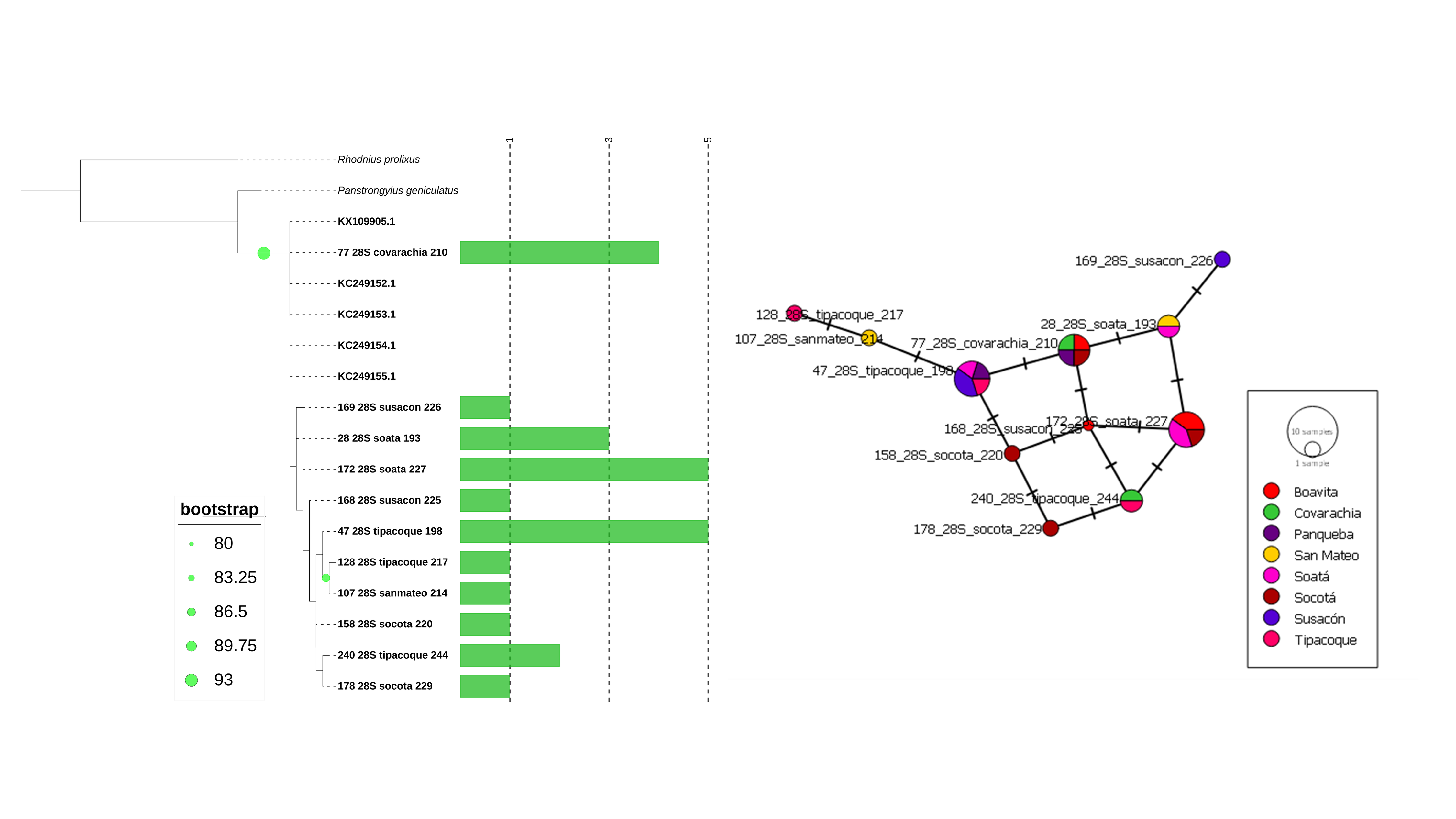

Supplement: S2 Fig — Green bars on the left indicate the number of haplotypes each sequence had. (TIF) [file pntd.0010534.s002.tif]

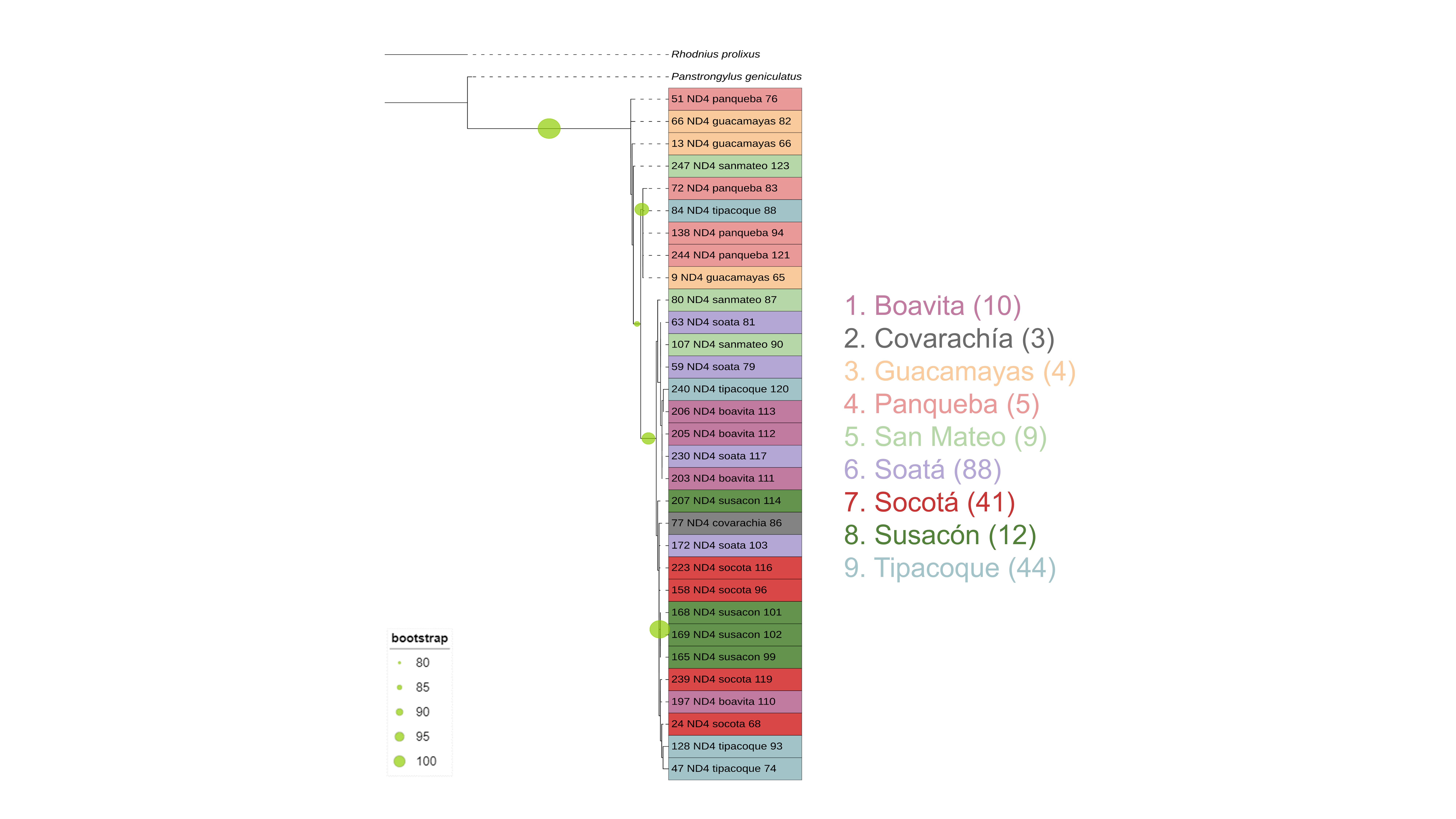

Supplement: S3 Fig — The tree shows the evolutionary relationships among the sequences obtained in this study. Legend follows the same color code per municipality used for the phylogenetic reconstruction. (TIF) [file pntd.0010534.s003.tif]

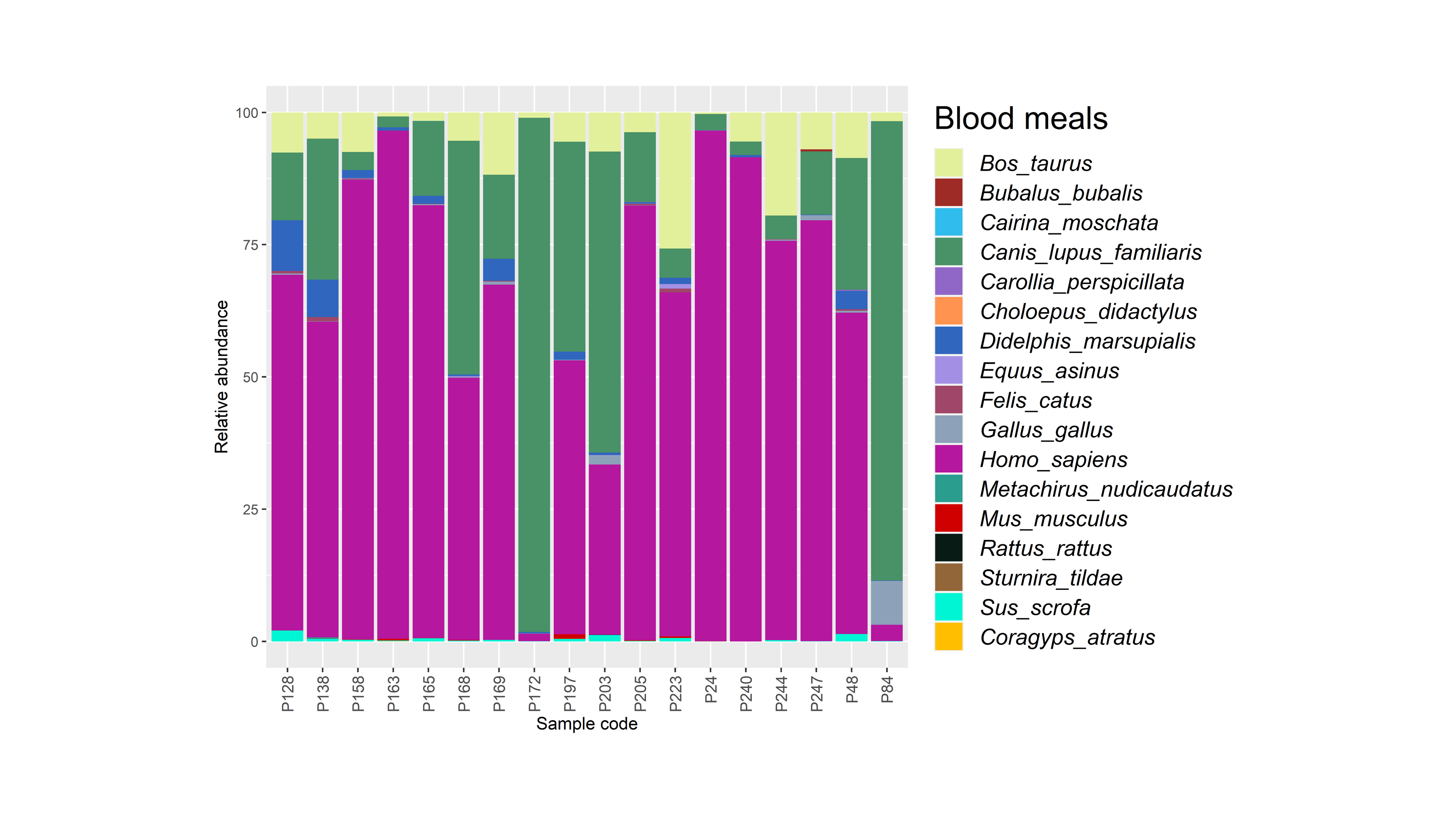

Supplement: S4 Fig — (TIF) [file pntd.0010534.s004.tif]
