# Supplementary material for: Estimating the genetic structure of Triatoma dimidiata (Hemiptera: Reduviidae) and the transmission dynamics of Trypanosoma cruzi in Boyacá, eastern Colombia
Source: PLoS Negl Trop Dis. 2022 Jul 11;16(7):e0010534. doi: 10.1371/journal.pntd.0010534 (PMC9302734; doi:10.1371/journal.pntd.0010534)
Supplement: S1 Table — (DOCX) [file pntd.0010534.s005.docx]

**Table S1. Summary table that contains information from the 229 triatomines analyzed.**

| **Sample code** | **Stage** | **Surveillance program** | **Municipality** | **Ecotope** | **Month of collection** |
| --- | --- | --- | --- | --- | --- |
| 1 | Adult | Passive | Soatá | Intradomicile | July |
| 2 | Adult | Passive | Soatá | Intradomicile | July |
| 3 | Nymph | Passive | Soatá | Intradomicile | May |
| 4 | Adult | Passive | San Mateo | Peridomicile | December |
| 5 | Adult | Passive | Soatá | Intradomicile | June |
| 6 | Adult | Passive | Tipacoque | Intradomicile | NA |
| 9 | Nymph | Passive | Guacamayas | Intradomicile | December |
| 10 | Adult | Passive | Soatá | Intradomicile | January |
| 11 | Nymph | Active | Socota | Intradomicile | March |
| 12 | Nymph | Passive | Soatá | Intradomicile | June |
| 13 | Adult | Passive | Guacamayas | Intradomicile | February |
| 14 | Adult | Passive | Susacón | Intradomicile | May |
| 15 | Adult | Passive | Tipacoque | Intradomicile | January |
| 17 | Adult | Passive | Soatá | Intradomicile | February |
| 18 | Adult | Passive | Soatá | Intradomicile | February |
| 19 | Adult | Passive | Tipacoque | Intradomicile | July |
| 20 | Adult | Passive | Tipacoque | Intradomicile | March |
| 21 | Adult | Passive | Soatá | Intradomicile | July |
| 22 | Adult | Passive | Tipacoque | Intradomicile | May |
| 23 | Nymph | Passive | Tipacoque | Intradomicile | January |
| 24 | Adult | Passive | Socota | Intradomicile | May |
| 25 | Adult | Passive | Socota | Intradomicile | May |
| 26 | Adult | Passive | Socota | Intradomicile | May |
| 27 | Nymph | Passive | Soatá | Intradomicile | March |
| 28 | Adult | Passive | Soatá | Intradomicile | January |
| 29 | Adult | Passive | Soatá | Intradomicile | April |
| 30 | Adult | Passive | San Mateo | Intradomicile | April |
| 31 | Adult | Passive | San Mateo | Intradomicile | April |
| 32 | Nymph | Passive | Socota | Intradomicile | March |
| 33 | Adult | Passive | Soatá | Peridomicile | July |
| 34 | Adult | Passive | Soatá | Intradomicile | April |
| 35 | Adult | Passive | Soatá | Intradomicile | April |
| 36 | Adult | Passive | Soatá | Intradomicile | July |
| 37 | Nymph | Passive | Tipacoque | Intradomicile | May |
| 38 | Adult | Passive | Tipacoque | Intradomicile | May |
| 39 | Nymph | Passive | Tipacoque | Intradomicile | May |
| 40 | Nymph | Passive | Tipacoque | Intradomicile | May |
| 42 | Adult | Passive | Soatá | Intradomicile | August |
| 43 | Adult | Passive | Tipacoque | Intradomicile | January |
| 44 | Adult | Passive | Tipacoque | Intradomicile | February |
| 45 | Adult | Passive | Soatá | Intradomicile | April |
| 46 | Adult | Active | Socota | Intradomicile | March |
| 47 | Adult | Passive | Tipacoque | Intradomicile | February |
| 48 | Adult | Passive | Covarachía | Intradomicile | March |
| 49 | Adult | Passive | Soatá | Intradomicile | July |
| 50 | Adult | Passive | Tipacoque | Intradomicile | June |
| 51 | Adult | Active | Panqueba | Intradomicile | May |
| 52 | Adult | Passive | Soatá | Intradomicile | July |
| 53 | Adult | Active | Socota | Intradomicile | March |
| 57 | Adult | Passive | Socota | Intradomicile | April |
| 58 | Nymph | Passive | Socota | Intradomicile | April |
| 59 | Adult | Passive | Soatá | Intradomicile | April |
| 60 | Adult | Passive | Soatá | Intradomicile | April |
| 61 | Nymph | Passive | Soatá | Intradomicile | April |
| 62 | Nymph | Passive | Soatá | Intradomicile | April |
| 63 | Adult | Passive | Soatá | Intradomicile | May |
| 64 | Nymph | Passive | Tipacoque | Intradomicile | July |
| 65 | Adult | Passive | Soatá | Intradomicile | July |
| 66 | Adult | Passive | Guacamayas | Intradomicile | May |
| 67 | Nymph | Passive | Susacón | Intradomicile | May |
| 68 | Adult | Passive | Soatá | Intradomicile | February |
| 69 | Adult | Passive | Soatá | Intradomicile | June |
| 70 | Adult | Passive | Tipacoque | Intradomicile | June |
| 71 | Nymph | Passive | Panqueba | Intradomicile | May |
| 72 | Adult | Passive | Panqueba | Intradomicile | May |
| 73 | Adult | Passive | Soatá | Intradomicile | May |
| 74 | Adult | Passive | Tipacoque | Intradomicile | March |
| 75 | Adult | Passive | Tipacoque | Intradomicile | June |
| 76 | Adult | Passive | Soatá | Intradomicile | January |
| 77 | Nymph | Passive | Covarachía | Intradomicile | February |
| 78 | Adult | Passive | Soatá | Intradomicile | February |
| 79 | Adult | Passive | Soatá | Intradomicile | February |
| 80 | Nymph | Passive | San Mateo | Peridomicile | February |
| 81 | Adult | Passive | Tipacoque | Intradomicile | May |
| 82 | Adult | Passive | Tipacoque | Intradomicile | May |
| 83 | Adult | Passive | Tipacoque | Intradomicile | May |
| 84 | Adult | Passive | Tipacoque | Intradomicile | May |
| 85 | Adult | Passive | Tipacoque | Intradomicile | May |
| 86 | Adult | Passive | Tipacoque | Intradomicile | January |
| 87 | Adult | Passive | Soatá | Intradomicile | February |
| 88 | Adult | Passive | Soatá | Intradomicile | February |
| 89 | Adult | Passive | Soatá | Intradomicile | February |
| 90 | Nymph | Passive | Tipacoque | Intradomicile | February |
| 91 | Adult | Passive | Panqueba | Peridomicile | March |
| 92 | Adult | Passive | Soatá | Intradomicile | NA |
| 93 | Adult | Passive | Soatá | Intradomicile | NA |
| 94 | Adult | Passive | Soatá | Intradomicile | NA |
| 95 | Adult | Passive | Soatá | Intradomicile | NA |
| 96 | Adult | Passive | Soatá | Intradomicile | July |
| 97 | Adult | Passive | Tipacoque | Intradomicile | January |
| 99 | Adult | Passive | Tipacoque | Intradomicile | April |
| 100 | Adult | Passive | Tipacoque | Intradomicile | April |
| 101 | Nymph | Passive | Tipacoque | Intradomicile | April |
| 102 | Adult | Passive | Socota | Intradomicile | April |
| 103 | Adult | Passive | Socota | Intradomicile | April |
| 104 | Adult | Passive | Tipacoque | Intradomicile | May |
| 105 | Adult | Passive | Soatá | Intradomicile | February |
| 106 | Nymph | Passive | Soatá | Intradomicile | February |
| 107 | Adult | Passive | San Mateo | Intradomicile | April |
| 108 | Adult | Passive | San Mateo | Intradomicile | April |
| 109 | Adult | Passive | San Mateo | Intradomicile | April |
| 110 | Adult | Passive | Soatá | Intradomicile | June |
| 111 | Adult | Passive | Soatá | Intradomicile | July |
| 112 | Adult | Passive | Soatá | Intradomicile | July |
| 113 | Adult | Passive | Soatá | Intradomicile | July |
| 114 | Adult | Passive | Soatá | Intradomicile | May |
| 115 | Adult | Passive | Tipacoque | Intradomicile | January |
| 116 | Adult | Passive | Tipacoque | Intradomicile | January |
| 117 | Adult | Passive | Soatá | Intradomicile | February |
| 118 | Adult | Passive | Soatá | Intradomicile | February |
| 121 | Adult | Passive | Soatá | Intradomicile | February |
| 122 | Nymph | Passive | Soatá | Intradomicile | February |
| 123 | Adult | Passive | San Mateo | Peridomicile | February |
| 124 | Adult | Passive | Tipacoque | Intradomicile | February |
| 125 | Adult | Active | Socota | Intradomicile | March |
| 128 | Adult | Passive | Tipacoque | Intradomicile | April |
| 129 | Adult | Passive | Soatá | Peridomicile | June |
| 130 | Adult | Passive | Guacamayas | Intradomicile | December |
| 131 | Adult | Passive | Tipacoque | Intradomicile | March |
| 132 | Adult | Passive | Soatá | Intradomicile | February |
| 133 | Adult | Passive | Soatá | Intradomicile | February |
| 134 | Adult | Passive | Soatá | Peridomicile | June |
| 136 | Nymph | Passive | Tipacoque | Intradomicile | May |
| 137 | Adult | Passive | Covarachía | Intradomicile | January |
| 138 | Adult | Passive | Panqueba | Intradomicile | April |
| 139 | Nymph | Active | Soatá | Intradomicile | January |
| 141 | Adult | Passive | Tipacoque | Intradomicile | April |
| 152 | Adult | Passive | Soatá | Intradomicile | April |
| 153 | Adult | Passive | Soatá | Intradomicile | April |
| 155 | Adult | Passive | Soatá | Peridomicile | July |
| 156 | Adult | Passive | Soatá | Intradomicile | July |
| 157 | Adult | Passive | Socota | Intradomicile | May |
| 158 | Adult | Passive | Socota | Intradomicile | May |
| 159 | Adult | Passive | Soatá | Peridomicile | August |
| 160 | Adult | Passive | Boavita | Peridomicile | December |
| 161 | Adult | Passive | Tipacoque | Intradomicile | February |
| 163 | Adult | Passive | Susacón | Intradomicile | April |
| 164 | Adult | Passive | Susacón | Intradomicile | April |
| 165 | Adult | Passive | Susacón | Intradomicile | April |
| 166 | Adult | Passive | Susacón | Intradomicile | April |
| 167 | Adult | Passive | Susacón | Intradomicile | April |
| 168 | Adult | Passive | Susacón | Intradomicile | April |
| 169 | Adult | Passive | Susacón | Intradomicile | April |
| 170 | Adult | Passive | Susacón | Intradomicile | April |
| 171 | Adult | Passive | Soatá | Intradomicile | May |
| 172 | Adult | Passive | Soatá | Intradomicile | May |
| 173 | Adult | Passive | Socota | Intradomicile | May |
| 174 | Adult | Passive | Socota | Intradomicile | May |
| 175 | Adult | Passive | Socota | Intradomicile | May |
| 176 | Adult | Passive | Socota | Intradomicile | May |
| 177 | Adult | Passive | Socota | Intradomicile | January |
| 178 | Adult | Passive | Socota | Intradomicile | January |
| 179 | Adult | Passive | Socota | Intradomicile | January |
| 180 | Adult | Passive | Socota | Intradomicile | January |
| 181 | Adult | Passive | Socota | Intradomicile | January |
| 182 | Adult | Passive | Socota | Intradomicile | January |
| 183 | Adult | Passive | Socota | Intradomicile | January |
| 184 | Adult | Passive | Socota | Intradomicile | January |
| 185 | Adult | Passive | Socota | Intradomicile | January |
| 186 | Adult | Passive | Socota | Intradomicile | January |
| 195 | Adult | Passive | Tipacoque | Intradomicile | December |
| 196 | Adult | Passive | Soatá | Intradomicile | January |
| 197 | Adult | Passive | Boavita | Intradomicile | September |
| 198 | Adult | Passive | Socota | Peridomicile | March |
| 199 | Nymph | Passive | Soatá | Intradomicile | November |
| 203 | Adult | Passive | Boavita | Intradomicile | December |
| 204 | Nymph | Passive | Boavita | Intradomicile | December |
| 205 | Adult | Passive | Boavita | Intradomicile | December |
| 206 | Nymph | Passive | Boavita | Intradomicile | December |
| 207 | Adult | Passive | Susacón | Peridomicile | October |
| 208 | Adult | Passive | Soatá | Intradomicile | August |
| 209 | Adult | Passive | Soatá | Peridomicile | August |
| 210 | Adult | Passive | Soatá | Intradomicile | September |
| 211 | Adult | Active | Tipacoque | Intradomicile | December |
| 212 | Adult | Active | Tipacoque | Intradomicile | December |
| 213 | Nymph | Active | Tipacoque | Intradomicile | December |
| 214 | Nymph | Active | Tipacoque | Intradomicile | December |
| 215 | Adult | Passive | Soatá | Intradomicile | September |
| 216 | Adult | Passive | Boavita | Intradomicile | July |
| 217 | Adult | Passive | Boavita | Intradomicile | July |
| 218 | Adult | Passive | Boavita | Intradomicile | July |
| 219 | Adult | Passive | Boavita | Intradomicile | July |
| 220 | Nymph | Active | Tipacoque | Intradomicile | December |
| 221 | Nymph | Active | Tipacoque | Intradomicile | December |
| 222 | Nymph | Active | Tipacoque | Intradomicile | December |
| 223 | Adult | Passive | Socota | Intradomicile | September |
| 224 | Adult | Passive | Socota | Intradomicile | September |
| 225 | Nymph | Passive | Socota | Intradomicile | September |
| 226 | Nymph | Passive | Socota | Intradomicile | September |
| 227 | Adult | Passive | Soatá | Intradomicile | September |
| 228 | Adult | Passive | Soatá | Intradomicile | December |
| 229 | Adult | Passive | Soatá | Intradomicile | April |
| 230 | Adult | Passive | Soatá | Intradomicile | April |
| 231 | Adult | Passive | Socota | Intradomicile | October |
| 232 | Adult | Passive | Socota | Intradomicile | October |
| 233 | Adult | Passive | Soatá | Intradomicile | September |
| 234 | Adult | Passive | Tipacoque | Intradomicile | November |
| 235 | Adult | Passive | Tipacoque | Intradomicile | November |
| 236 | Adult | Passive | Tipacoque | Intradomicile | November |
| 237 | Adult | Passive | Soatá | Intradomicile | August |
| 238 | Adult | Passive | Soatá | Intradomicile | August |
| 239 | Adult | Passive | Socota | Intradomicile | August |
| 240 | Adult | Passive | Tipacoque | Intradomicile | January |
| 241 | Adult | Passive | Soatá | Intradomicile | August |
| 242 | Adult | Passive | Socota | Intradomicile | August |
| 243 | Adult | Passive | Socota | Intradomicile | August |
| 244 | Nymph | Passive | Panqueba | Intradomicile | July |
| 245 | Adult | Passive | Soatá | Intradomicile | November |
| 246 | Adult | Passive | Socota | Intradomicile | March |
| 247 | Adult | Passive | San Mateo | Intradomicile | June |
| 248 | Adult | Passive | Soatá | Intradomicile | December |
| 249 | Adult | Passive | Tipacoque | Intradomicile | November |
| 250 | Adult | Passive | Soatá | Peridomicile | September |
| 251 | Adult | Passive | Soatá | Intradomicile | August |
| 252 | Adult | Passive | Susacón | Intradomicile | October |
| 253 | Adult | Passive | Soatá | Intradomicile | November |
| 254 | Adult | Passive | Soatá | Intradomicile | November |
| 255 | Adult | Passive | Soatá | Intradomicile | November |
| 256 | Adult | Passive | Soatá | Intradomicile | November |
| 257 | Adult | Passive | Soatá | Intradomicile | November |
| 258 | Adult | Passive | Soatá | Intradomicile | November |
| 259 | Adult | Passive | Soatá | Intradomicile | November |
| 260 | Adult | Passive | Soatá | Intradomicile | November |
| 261 | Adult | Passive | Socota | Intradomicile | January |
| 262 | Nymph | Passive | Socota | Intradomicile | January |
| 263 | Adult | Passive | Socota | Intradomicile | January |
| 264 | Adult | Passive | Socota | Intradomicile | January |
| 265 | Adult | Passive | Socota | Intradomicile | January |
| 266 | Adult | Passive | Socota | Intradomicile | January |
