# Supplementary material for: Estimating the genetic structure of Triatoma dimidiata (Hemiptera: Reduviidae) and the transmission dynamics of Trypanosoma cruzi in Boyacá, eastern Colombia
Source: PLoS Negl Trop Dis. 2022 Jul 11;16(7):e0010534. doi: 10.1371/journal.pntd.0010534 (PMC9302734; doi:10.1371/journal.pntd.0010534)
Supplement: S2 Table — (DOCX) [file pntd.0010534.s006.docx]

**S2 Table. GenBank accession codes for the molecular markers used in the phylogenetic analyses.**

| **Species** | **ND4** | **cytb** | **28S (D2 domain)** |
| --- | --- | --- | --- |
| *Triatoma dimidiata* | KC489292- KC489463 [1] | KT998309, KT998310, KT998332 [3] | KC249152- KC249155* [4], KX109905.1 [2] |
| *Rhodnius prolixus* | NC_050328.1 | NC_050328.1 | AF435862 |
| *Panstrongylus geniculatus* | MK830021 | MK829943 | KX109907 |

ITS-2 marker does not appear in phylogenetic analyses because haplotype network showed a pie-chart pattern (only one haplotype), and no SNPs.

*GenBank sequences from Central America

References

1. Pfeiler E, Bitler B, Ramsey J, Palacios-Cardiel C, Markow T. Genetic variation, population structure, and phylogenetic relationships of Triatoma rubida and T. recurva (Hemiptera: Reduviidae: Triatominae) from the Sonoran Desert, insect vectors of the Chagas’ disease parasite Trypanosoma cruzi. Mol. Phylogenet. Evol. 2006; 41, 209–221.
2. Di Iorio O, Gürtler R. Seasonality and Temperature-Dependent Flight Dispersal of Triatoma infestans (Hemiptera: Reduviidae) and Other Vectors of Chagas Disease in Western Argentina, Journal of Medical Entomology, Volume 54, Issue 5. 2017, Pages 1285–1292.
3. Boyacá Climate Weather Averages. Available in: <https://www.worldweatheronline.com/Boyacá-weather-averages/Boyacá-department/co.aspx>
4. Rincón-Galvis H, Urbano P, Hernández C, Ramírez J, Florin D. Temporal Variation of the Presence of Rhodnius prolixus (Hemiptera: Reduviidae) into Rural Dwellings in the Department of Casanare, Eastern Colombia. J Med Entomol. 2020;57(1):173–80.
