# Supplementary material for: Estimating the genetic structure of Triatoma dimidiata (Hemiptera: Reduviidae) and the transmission dynamics of Trypanosoma cruzi in Boyacá, eastern Colombia
Source: PLoS Negl Trop Dis. 2022 Jul 11;16(7):e0010534. doi: 10.1371/journal.pntd.0010534 (PMC9302734; doi:10.1371/journal.pntd.0010534)
Supplement: S3 Table — (DOCX) [file pntd.0010534.s007.docx]

**Table S3. GenBank accession codes of the sequences used for the construction of the reference DTUs dataset for BLAST similarity search.**

| **DTU/Genotype** | **Accession code** |
| --- | --- |
| Strain CG C TcIa Caqueta | AM259467.1 |
| Strain EMA TcIa Boyacá | EU344772.1 |
| Strain SN6 C TcIa Magdalena | AM259471.1 |
| Strain Mg10 C TcIb Magdalena | AM259477.1 |
| Strain X150C TcIb Boyacá | EU127307.1 |
| Strain Td3C TcIb Boyacá | EU127312.1 |
| Strain G11C TcIb Boyacá | EU127315.1 |
| Strain X380 C TcIc Boyacá | AM259472.1 |
| Strain DM28 C TcId Tolima | AM259470.1 |
| Strain Dm11C TcId Tolima | EU127304.1 |
| Strain Col 108 Chile TcIe | GU903125.1 |
| Strain Sp Guayacan Chile TcIe | GU903141.1 |
| Strain Gaj29 cl6 Chile TcIe | GU903129.1 |
| Strain Til70 cl7 Chile TcId+TcIe | GU903146.1 |
| Strain Tu18 TcII | AY367125.1 |
| Strain Esmeraldo TcII | ANOX01015751.1 |
| Strain M6241 TcIII | AF050522.1 |
| Strain 92122102r TcIV | AY367124.1 |
| Strain SC43 TcV | AY367127.1 |
| Strain CL TcVI | U57984.1 |
| Strain TCC 2477 Tcbat | KT305884.1 |
