# Supplementary material for: Estimating the genetic structure of Triatoma dimidiata (Hemiptera: Reduviidae) and the transmission dynamics of Trypanosoma cruzi in Boyacá, eastern Colombia
Source: PLoS Negl Trop Dis. 2022 Jul 11;16(7):e0010534. doi: 10.1371/journal.pntd.0010534 (PMC9302734; doi:10.1371/journal.pntd.0010534)
Supplement: S4 Table — (DOCX) [file pntd.0010534.s008.docx]

**Table S4. Summary table that contains sequences samples crossed information.**

| **Sample code** | **Stage** | **Municipality** | **Ecotope** | **qPCR** | **Parasite load** | **Sequencing type (Mini-exon)** | **TcI genotype** | **Sequencing type (12S)** | **Most common blood meals** |
| --- | --- | --- | --- | --- | --- | --- | --- | --- | --- |
| 24 | Adult | Socotá | Intradomicile | Pos | 78,118,912 | Illumina Hi-seq | TcIb, TcId | Illumina Hi-seq | *Homo sapiens sapiens, Canis lupus familiaris* |
| 48 | Adult | Covarachía | Intradomicile | Pos | 29,311,420 | Illumina Hi-seq | TcIb, TcId | Illumina Hi-seq | *Homo sapiens sapiens, Canis lupus familiaris, Bos taurus* |
| 63 | Adult | Soatá | Intradomicile | Pos | 85,356,448 | Illumina Hi-seq | TcIa, TcIb, TcId | Sanger | *Homo sapiens sapiens* |
| 66 | Adult | Guacamayas | Intradomicile | Pos | 11,478,070 | Illumina Hi-seq | TcIb, TcId, TcIc | Sanger | *Canis lupus familiaris* |
| 72 | Adult | Panqueba | Intradomicile | Pos | 84,524,432 | Illumina Hi-seq | TcIb | Sanger | *Homo sapiens sapiens* |
| 84 | Adult | Tipacoque | Intradomicile | Pos | 61,558,340 | Sanger | TcIb | Illumina Hi-seq | *Canis lupus familiaris, Gallus gallus, Homo sapiens sapiens* |
| 107 | Adult | San Mateo | Intradomicile | Pos | 90,824,192 | Illumina Hi-seq | TcId, TcIb, TcIa, TcIc | Sanger | *Homo sapiens sapiens* |
| 128 | Adult | Tipacoque | Intradomicile | Pos | 354,484,800 | Illumina Hi-seq | TcIb, TcId, TcIa | Illumina Hi-seq | *Homo sapiens sapiens, Canis lupus familiaris, Didelphis marsupialis, Bos taurus* |
| 138 | Nymph | Panqueba | Intradomicile | Pos | 9,731,532 | Illumina Hi-seq | TcIb, TcIa, TcId | Illumina Hi-seq | *Homo sapiens sapiens, Canis lupus familiaris, Didelphis marsupialis, Bos taurus* |
| 158 | Adult | Socotá | Intradomicile | Pos | 9,095 | Sanger | TcIb | Illumina Hi-seq | *Homo sapiens sapiens, Bos taurus, Canis lupus familiaris* |
| 163 | Adult | Susacón | Intradomicile | Pos | 17,441 | Illumina Hi-seq | TcIb, TcId, TcIa | Illumina Hi-seq | *Homo sapiens sapiens, Canis lupus familiaris* |
| 165 | Adult | Susacón | Intradomicile | Pos | 37,680 | Sanger | TcIb | Illumina Hi-seq | *Homo sapiens sapiens, Canis lupus familiaris* |
| 168 | Adult | Susacón | Intradomicile | Pos | 30,144,654 | Illumina Hi-seq | TcIb, TcId, TcIa | Illumina Hi-seq | *Homo sapiens sapiens, Canis lupus familiaris, Bos taurus* |
| 169 | Adult | Susacón | Intradomicile | Pos | 131,876 | Illumina Hi-seq | TcId, TcIb, TcIa, TcIc | Illumina Hi-seq | *Homo sapiens sapiens, Canis lupus familiaris, Bos taurus, Didelphis marsupialis* |
| 172 | Adult | Soatá | Intradomicile | Pos | 878,414 | Illumina Hi-seq | TcIb, TcId, TcIa | Illumina Hi-seq | *Canis lupus familiaris, Homo sapiens sapiens, Bos taurus* |
| 178 | Adult | Socotá | Intradomicile | Pos | 12,074,747 | Illumina Hi-seq | TcId, TcIb, TcIa, TcIc | Sanger | *Homo sapiens sapiens* |
| 197 | Adult | Boavita | Intradomicile | Pos | 11,909,588 | Illumina Hi-seq | TcIb, TcId, TcIc | Illumina Hi-seq | *Homo sapiens sapiens, Canis lupus familiaris, Bos taurus* |
| 203 | Adult | Boavita | Intradomicile | Pos | 906 | Sanger | TcIb | Illumina Hi-seq | *Canis lupus familiaris, Homo sapiens sapiens, Bos taurus* |
| 205 | Adult | Boavita | Intradomicile | Pos | 978 | Illumina Hi-seq | TcIb, TcId | Illumina Hi-seq | *Homo sapiens sapiens, Canis lupus familiaris, Bos taurus* |
| 206 | Nymph | Boavita | Intradomicile | Pos | 67,831,832 | Illumina Hi-seq | TcIb, TcId | Sanger | *Homo sapiens sapiens* |
| 207 | Adult | Susacón | Peridomicile | Pos | 36,239,480 | Sanger | TcIb | Sanger | *Homo sapiens sapiens* |
| 223 | Adult | Socotá | Intradomicile | Pos | 23,447,950 | Illumina Hi-seq | TcIb, TcId, TcIa | Illumina Hi-seq | *Homo sapiens sapiens, Bos taurus, Canis lupus familiaris* |
| 230 | Adult | Soatá | Intradomicile | Pos | 16,872,330 | Illumina Hi-seq | TcIb, TcId, TcIa, TcIc | Sanger | *Homo sapiens sapiens* |
| 239 | Adult | Socotá | Intradomicile | Pos | 6,761 | Sanger | TcIb | Sanger | *Canis lupus familiaris* |
| 240 | Adult | Tipacoque | Intradomicile | Pos | 5,646,003 | Illumina Hi-seq | TcIb, TcId | Illumina Hi-seq | *Homo sapiens sapiens, Bos taurus, Canis lupus familiaris* |
| 244 | Nymph | Panqueba | Intradomicile | Pos | 19,033,480 | Illumina Hi-seq | TcIb, TcId | Illumina Hi-seq | *Homo sapiens sapiens, Bos taurus, Canis lupus familiaris* |
| 247 | Adult | San Mateo | Intradomicile | Pos | 48,741,376 | Illumina Hi-seq | TcIb, TcId, TcIa | Illumina Hi-seq | *Homo sapiens sapiens, Bos taurus, Canis lupus familiaris* |
