# Supplementary material for: Estimating the genetic structure of Triatoma dimidiata (Hemiptera: Reduviidae) and the transmission dynamics of Trypanosoma cruzi in Boyacá, eastern Colombia
Source: PLoS Negl Trop Dis. 2022 Jul 11;16(7):e0010534. doi: 10.1371/journal.pntd.0010534 (PMC9302734; doi:10.1371/journal.pntd.0010534)
Supplement: S5 Table — (DOCX) [file pntd.0010534.s009.docx]

**Table S5. Summary of entomological indices per municipality.**

| **Municipality** | **Collection type** | **Examined houses** | **Infested houses** | **Examined triatomines** | **Infected triatomines** | **Nymphs** | **Infected nymphs** | **Nymphs inside** | **Infestation (%)** | **Colonization (%)** | **Density** | **Crowding** | **Infection (%)** |
| --- | --- | --- | --- | --- | --- | --- | --- | --- | --- | --- | --- | --- | --- |
| Boavita | Ps | 3 | 3 | 9 | 9 | 1 | 1 | 1 | 100 | 33.3 | 3 | 3 | 100 |
| Covarachía | Ps | 3 | 2 | 3 | 2 | 1 | 0 | 1 | 67 | 50 | 1 | 1.5 | 66.7 |
| Guacamayas | Ps | 4 | 2 | 4 | 2 | 1 | 0 | 1 | 50 | 50 | 1 | 2 | 50 |
| Panqueba | Ps | 4 | 3 | 4 | 3 | 2 | 1 | 2 | 75 | 66.7 | 1.0 | 1.3 | 75 |
|  | As | 1 | 0 | 1 | 0 | 0 | 0 | 0 | - | - | 1.0 | - | 0.0 |
| San Mateo | Ps | 3 | 3 | 6 | 4 | 0 | 0 | 0 | 100 | - | 2 | 2 | 66.7 |
| Soatá | Ps | 52 | 35 | 81 | 55 | 8 | 3 | 7 | 67.3 | 20 | 1.6 | 2.3 | 67.9 |
|  | As | 1 | 1 | 1 | 1 | 1 | 1 | 1 | 100 | 100 | 1.0 | 1.0 | 100 |
| Socotá | Ps | 12 | 10 | 40 | 35 | 5 | 3 | 2 | 83.3 | 20 | 3.3 | 4 | 87.5 |
|  | AS | 4 | 0 | 4 | 0 | 1 | 0 | 1 | - | - | 1.0 | - | 0.0 |
| Susacón | Ps | 4 | 2 | 12 | 9 | 1 | 0 | 1 | 50 | 50 | 3.0 | 6 | 75 |
| Tipacoque | Ps | 32 | 20 | 44 | 25 | 8 | 2 | 7 | 62.5 | 35 | 1.4 | 2.2 | 56.8 |
|  | AS | 2 | 2 | 7 | 7 | 5 | 5 | 2 | 100.0 | 100 | 3.5 | 3.5 | 100 |
| Total |  | 125 | 83 | 216 | 152 | 34 | 16 | 26 |  |  |  |  |  |

Ps = passive surveillance; As = active surveillance
